# Experimental Evidences on Magnetism-Covalent Bonding Interplay in Structural Properties of Solids and during Chemisorption

**DOI:** 10.3390/ijms25031793

**Published:** 2024-02-01

**Authors:** Chiara Biz, Jose Gracia, Mauro Fianchini

**Affiliations:** MagnetoCat SL, Calle General Polavieja 9, 3 Izq, 03012 Alicante, Spain; chiara.biz@magnetocat.com

**Keywords:** magnetism, covalent bonding, magnetic methods, heterogeneous catalysis, chemisorption processes

## Abstract

Valence electrons are one of the main players in solid catalysts and in catalytic reactions, since they are involved in several correlated phenomena like chemical bonding, magnetism, chemisorption, and bond activation. This is particularly true in the case of solid catalysts containing *d*-transition metals, which exhibit a wide range of magnetic phenomena, from paramagnetism to collective behaviour. Indeed, the electrons of the outer *d*-shells are, on one hand, involved in the formation of bonds within the structure of a catalyst and on its surface, and, on the other, they are accountable for the magnetic properties of the material. For this reason, the relationship between magnetism and heterogeneous catalysis has been a source of great interest since the mid-20th century. The subject has gained a lot of attention in the last decade, thanks to the orbital engineering of quantum spin–exchange interactions and to the widespread application of external magnetic fields as boosting tools in several catalytic reactions. The topic is discussed here through experimental examples and evidences of the interplay between magnetism and covalent bonding in the structure of solids and during the chemisorption process. Covalent bonding is discussed since it represents one of the strongest contributions to bonds encountered in materials.

## 1. Introduction

Catalytic events occur through breaking and forming bonds over the surface of a catalyst in heterogeneous catalysis. The nature of such bonds is the result of several combined contributions (e.g., Coulomb, van der Waals, exchange, ionic …) which in turn depend on the electronic structure of the catalyst [1]. The majority of the catalysts of industrial interest are composed of elements with almost filled or fully filled 3*d*-, 4*d*-, or 5*d*-orbitals [1,2], which are also known to display magnetic properties [3,4]. Indeed, state-of-the-art catalysts such as platinum (Pt), iridium (Ir), palladium (Pd), iridium dioxide (IrO_2_), and ruthenium dioxide (RuO_2_) are classified as paramagnetic [5,6]. Paramagnetism occurs only in materials with unpaired electrons [3,7], and it is an expression of a deeper and multifaceted phenomenon known as magnetism (other expressions of this phenomenon are superparamagnetism, ferromagnetism, ferrimagnetism, and antiferromagnetism) [4]. Readers can find background information on magnetism and the magnetic properties of solid catalysts in the literature [8,9].

In the case of *d*-transition metal elements, their paramagnetic properties arise from the electrons of the outer *d*-shells [10,11]—the very same outer *d*-electrons that also participate in the formation of chemical bonds in materials and during catalytic reactions [11]. The relationship between magnetism and heterogeneous catalysis has been a debated topic in the scientific community since the middle of the 20th century. P.W. Selwood wrote in his work titled “*Magnetism and catalysis*” (1946) that the most active elements in catalysis, the *d*-transition metal elements, also exhibit intriguing magnetic properties, provided that not all chemical processes depend on magnetism [12]. A few years later, D.A. Dowden (1950) reported that a decrement in paramagnetism or ferromagnetism is observed for some metals during the chemisorption process. This decrement should be taken into account to provide a more complete description of this chemical event [1]. Later, R.J.H. Voorhoeve (1974) discussed whether magnetic ordering can play a role in the elementary steps of catalytic reactions in his work titled “*Experimental Relationships between Catalysis and Magnetism*” [13]. As a final example, J.T. Richardson suggested in 1978 that maybe the property that makes solids paramagnetic (i.e., the presence of unpaired electrons) is the same property that also allows for the formation of bonds during chemisorption processes and the exchange of electrons during redox reactions [14]. This topic is still of modern interest since in the past few years, compositions containing magnetic 3*d*-transition metals as catalysts have been tested [15,16,17] and external magnetic fields (coupled or not with magnetic compositions) have been experimentally applied as a way to boost catalytic performances during catalytic reactions, particularly in oxygen catalysis [9,18,19]. Thus, in this work, we discuss the relationship between magnetism and heterogeneous catalysis through the interplay between magnetism and covalent bonding in the structure of materials and during the chemisorption process. Experimental examples and then more specific experimental evidences will be provided on this interplay, which involves magnetism in heterogeneous catalysis. Covalent bonds are discussed since they represent one of the strongest chemical binding contributions encountered in solids and in catalysis [20,21].

## 2. Discussion

Bonds in solids are commonly classified as ionic, covalent, metallic, or molecular [20,21,22]. The valence electrons in ionic bonds undergo heteropolar binding which creates opposite charges that become subjected to an electrostatic interaction among themselves. In contrast, the valence electrons in covalent bonds undergo homopolar sharing among the involved atoms by forming electron pairs that are localized in the internuclear area. In metallic bonding, the valence electrons are delocalized among the atomic cores and defined as nondirectional bonds. Dispersive interactions (e.g., hydrogen bonds, van der Waals interactions, hydrophobic interactions, London dispersion forces, and dipole–dipole interactions) are responsible for the formation of these so-called molecular bonds by inducing dipole moments among neutral atoms in materials with closed-shell configurations (e.g., noble gasses at extremely low temperatures and crystals of organic molecules) [20,21]. These dispersive interactions are attractive forces whose existence does not depend on any charge density overlap between two (or more) atoms [20]. Nevertheless, the real description of a chemical bond in materials is, more often than not, the result of all these types of contributions, but when one contribution is predominant, the materials can be categorized into four different groups, as shown in Table 1.

The strongest bonds encountered in materials are ionic and covalent bonds (see Table 1), which are also claimed to be the most common types of bonding involved in the control of the rate of the reaction during catalytic processes [1]. Nonetheless, one should not disregard the contributions of the other types of bonding in catalytic processes. To our knowledge, it is nearly impossible to separate bonding contributions in experiments. Nonetheless, the experimental quantification of the bond strength in the case of ionic crystals, for instance, can be carried out by descriptors like Madelung constants, which provide a measure of the electrostatic energy that binds the ions. For example, the Madelung constants of wurtzite (ZnS, M^2+^ X^2−^ ion type, hexagonal), 1.64132, and of zinc oxide (ZnO, M^2+^ X^2−^ ion type, hexagonal), 1.4985 [23], indicate that a higher amount of energy is required to break the crystal lattice of the former material because of the strong ionic contribution in its bonds. The separation and quantification of covalent, ionic, and non-covalent contributions in chemical bonds, in solid structures, and during catalytic events are more approachable via computational techniques and post-wavefunction analyses, like Atoms in Molecules (AIM) [24], NBO, RDG, DORI, and others.

### 2.1. On the Interplay between Magnetism and Covalent Bonding

Covalent bonds imply the sharing of valence electrons with opposite spins between atoms and thus the concentration of valence electron density between the nuclei via orbital overlapping. Covalent bonding is directional and strong, and is responsible for the structural stability, high melting points, low atomic coordination number, and poor conduction properties of covalent solids [20,21] (see Table 1 for some examples). Nevertheless, most solid catalysts are composted of *d*-transition metal elements whose outer *d*-electrons are also responsible for their paramagnetic and magnetic properties. The most common industrially employed, active, and state-of-the-art solid catalysts in heterogeneous catalysis nowadays contain transition metal elements, such as iron (Fe), cobalt (Co), nickel (Ni), ruthenium (Ru), molybdenum (Mo), palladium (Pd), rhodium (Rh), tungsten (W), platinum (Pt), and iridium (Ir). Table 2 shows several examples of these compositions employed in industrially relevant catalytic transformations and the operating conditions in which they are employed. All these solid catalysts possess well-known diverse magnetic properties that range from paramagnetism to collective magnetism. Some remarkable examples include the use of ferromagnetic platinum/cobalt (Pt/Co) alloys as ORR (oxygen reduction reaction) catalysts in commercially available fuel cells for electric vehicles [15,25] and state-of-the-art OER (oxygen evolution reaction) catalysts, like paramagnetic iridium dioxide (IrO_2_) [6,26] and antiferromagnetic ruthenium dioxide (RuO_2_) (with a Néel temperature up to ~300 K) [26,27,28]. Moreover, in the last few years, paramagnetic heterogeneous materials, in particular, ferromagnetic ones, have once more become the target of extensive studies on the influence of external applied magnetic fields in catalysis [18].

Only a few diamagnetic transition metals (e.g., copper (Cu), silver (Ag), zinc (Zn), and mercury (Hg)) [34] and diamagnetic oxides (e.g., aluminium oxide (Al_2_O_3_), zinc oxide (ZnO), and magnesium oxide (MgO)) [34] are industrially employed in catalytic reactions, as displayed in Table 2 [2,35]. However, the former are generally not claimed to be the most active/selective solid catalysts (e.g., in hydrogenation reactions with molecular hydrogen (H_2_) and hydrocarbons) [2], while the latter are commonly used as supporting materials in heterogeneous processes [35]. For example, copper-based catalysts (e.g., copper/ chromic oxide (Cu/Cr_2_O_3_) and copper/zinc oxide (Cu/ZnO)) are utilized in hydrogenation reactions under mild conditions [36,37], while silver-based materials (e.g., rhenium/cesium/silver/alumina (Re/Cs/Ag/Al_2_O_3_) and alkaline-metal-promoted supported silver/alumina (Ag/Al_2_O_3_)) are employed in the production of formaldehyde (CH_2_O) from methanol (CH_3_OH) [38] and in the oxidation of ethene (H_2_C=CH_2_) [39]. Materials with all paired *d*-electrons are supposed to be mediocre solid catalysts [2], and, interestingly, under the operating catalytic conditions, the active catalytic species are commonly reported to be oxidized forms of the diamagnetic metal precursors (e.g., copper oxides (CuO_x_) [33] and silver oxides (AgO_x_) [39]), which, most likely, cease to be diamagnetic. Diamagnetic elements of group eleven (copper (Cu), silver (Ag), and gold (Au)) and twelve (zinc (Zn), cadmium (Cd), and mercury (Hg)) in the periodic table possess a completely filled *d*-shell (e.g., Cu: [Ar] 3*d*^10^4*s*^1^ and Zn: [Ar] 3*d*^10^4*s*^2^). Such *d*-electrons are less prone to participate in chemical bonding since completely filled *d*-orbitals are less spatially extended than partially filled *d*-ones [40]. Thus, the presence of unpaired *d*-electrons in the valence shell of a solid catalyst is a conditio sine qua non to allow for the occurrence of catalytic processes through chemisorption [2]. The electronic ground state of a solid catalyst, together with its structure, correlates with its catalytic performance [1]. Table 3 summarizes the types and strengths of the energetic contributions of the Hamiltonian operator ($\hat{H}$) in the n*d*-series [7,41], in the absence of an applied external magnetic field.

The major energy contributions that affect the electronic ground state of *d*-electron orbitals are as follows: the electron–electron Coulomb repulsions ($V_{e^{-}+e^{-}}^{Coul}$), the crystal field potential ($V_{L}$), and the spin–orbit interaction ($V_{s-o}$). The different strengths and influences of these three energy contributions on 3*d*-, 4*d*-, and 5*d*-electron orbitals affect the electronic ground state of catalyst materials. Among the *d*-orbitals, 3*d*-orbitals are the least spatially extended ones. The higher energy contribution of the crystal field ($V_{L}$) in 4*d*- and 5*d*-orbitals is due to their higher radial extension than that of the 3*d*-ones, which maximizes the overlap with ligands, such as oxygen atoms in oxides. Another consequence of their large radial extension is the higher energy contribution of the spin–orbit coupling ($V_{s-o}$), which may produce a non-small orbital contribution to the total magnetic moment of 4*d*- or 5*d*-elements. Only the electron–electron Coulomb repulsion ($V_{e^{-}+e^{-}}^{Coul}$) is a minor energy contribution in 4*d*- and 5*d*- orbitals since, in a doubly occupied 4*d*-/5*d*-orbital, the on-site repulsion is lessened thanks to their higher orbital radial extension. Thus, the energy contributions of $V_{L}\;$ and $V_{s-o}$ should not be neglected in the treatment of the electronic ground state of 4*d*- and 5*d*-elements. In addition, none of the elements in the 4*d*- and 5*d*-series exhibit magnetically ordered states below a critical (or transition) temperature, unlike 3*d*-elements, which do so from chromium (Cr) to nickel (Ni) [3]. Only molybdenum (Mo), ruthenium (Ru), and rhodium (Rh) in the 4*d*-series; rhenium (Re), osmium (Os), and iridium (Ir) in the 5*d*-series; and their compounds are reported to exhibit magnetic properties [40]. These elements are also the most common 4*d*- and 5*d*-metals encountered in catalytic compositions [16,42,43,44]. Another important consequence of the higher orbital spatial extension of 4*d*- and 5*d*-elements is their higher covalency with respect to 3*d*-ones [40,45]. The presence of a higher covalent character in a material is generally associated with an increased chemical, thermal, and mechanical stability. It follows that solid catalysts containing 4*d*- and 5*d*-elements should be, at least in principle, more stable than 3*d*-based catalysts under the same catalytic operating conditions. The role of stability in catalysis is industrially crucial since it determines the lifetime of the solid catalysts in reactors [2]. Catalyst stability can also be improved via engineering magnetic interactions [28]. In this regard, the use of platinum ordered intermetallic compounds (Pt_1-x_M_x_ where M is Fe or Co) as ORR solid catalysts (see Table 2) is outstanding. These ordered intermetallic alloys experimentally show a more stable structure and a minor leaching of 3*d*-atoms in comparison with the corresponding disordered phase for the same chemical composition and nanoparticle size under the same catalytic operating conditions [9,46]. The chemical order is the most commonly used argument to explain the higher stability of these materials, even though one should also take into account their magnetic properties [9,46]. Indeed, these ordered intermetallic alloys are also characterized by the presence of a higher magnetic anisotropy than that of their corresponding disordered counterparts, which is responsible for their higher coercivity, higher Curie points, and their ability to retain magnetic properties at very small nanoparticle sizes [9]. These magnetic features are even more enhanced when the ratio between Pt and the 3*d*-element is 50:50 (i.e., L1_0_ PtFe, and PtCo intermetallic compositions) [9]. The engineering of magnetic interactions to improve the stability of solid catalysts is an example of the involvement of magnetism in heterogeneous catalysis.

### 2.2. Evidence of the Interplay between Magnetism and Covalent Bonding in Materials

The first scientist to explore the relationship between covalent bonding and magnetism was L. Pauling in his work “*The nature of the chemical bond*” (1931) [47]. A more complete treatment was then published by J. B. Goodenough in 1963, titled “*Magnetism and the chemical bond*”. This work added a special focus on magnetic oxides containing *3d*- and *4f*- elements, and it pointed out the importance of spin correlations in stabilizing chemical bonds in such materials [11]. Other authors like P.W. Selwood [12], J. Hubbard [48], J. Owen [49], B.C. Tofield [45], and P.W. Anderson [50] have contributed to this topic with their experimental and theoretical works.

Four main pieces of evidence on the interplay between covalency and magnetism in materials are listed below.

**Evidence 1.**  *The increment of covalency (i.e., the amounts and strengths of covalent bonds) is associated with the decrement in the magnetic susceptibility in materials [12]. The first scientist to report this effect was L. Pauling, who, in his second rule, stated “The spins of the electrons are opposed when the bond is formed, so that they cannot contribute to the paramagnetic susceptibility of the substance” [47].*

**Explanation 1.**  *Magnetic susceptibility (χ) measurements represent a preliminary characterization technique to investigate the magnetic behavior of materials [3,40,41]. In general, two main contributions to susceptibility can be identified: diamagnetic (χ_D_ < 0) and paramagnetic (χ_P_ > 0) susceptibilities [3,40]. The former is the contribution due to the interactions of paired electrons (orbital diamagnetism) with the external applied magnetic field (* $\vec{H}$ *) [3,40]. The latter is due to the interaction of unpaired electrons (i.e., spin and/or orbital angular momenta) with* $\vec{H}$ *[3,40]. Even though diamagnetism is a property of all matter [3,7], in the majority of materials, the diamagnetic contribution is smaller than the paramagnetic contribution [3,40]. Only some materials, typically insulators, with no unpaired electrons make a non-negligible diamagnetic contribution to χ [40]. Covalency, as well as diamagnetism, is also related to paired electrons. For instance, covalent solids such as diamond and Si are well-known diamagnetic materials (see Table 1) [20,21]. The increment in covalency, indicates that the solid is less “paramagnetic” and that it has a reduced sensitivity to the presence of an external applied magnetic field (i.e., a reduced total magnetic susceptibility).*

**Evidence 2.**  *An increased covalent character in chemical bonds can cause deviations in the magnetic moments of materials [12].* *It can also affect the spin–orbit interaction, particularly in the case of materials containing 4d- and 5d-transition metal elements [49].*

**Explanation 2.**  *The magnetic moment of atoms comprises two components: the spin and the orbital, which are associated with the spin (*$\vec{S}$*) and the orbital (*$\vec{L}$*) angular momenta [3,4]. The sum of*$\vec{S}$*and*$\vec{L}$*gives the total angular momentum (*$\vec{J)}$*, which is related to the total magnetic moment [3,4]. The presence of covalency in a material leads to a reduction in the orbital component (*$\vec{L}$*) of the total magnetic moments of the atoms [47,49]. If the covalent character in the bonds is very high, the orbital magnetic moments can completely disappear [47]. Theoretical explanations can be found in the works of* *L. Pauling [47],* *and J. Owen [49],* * while J. Hubbard [48]* *and B.C. Tofield [51,52]* *provided experimental proofs of this reduction through neutron diffraction techniques. For example, B. C. Tofield reported a reduction of 10.0 ± 0.5%, due to covalency, in the magnetic moment of G-type antiferromagnetic (AFM) lanthanum ferrite perovskite (LaFeO_3_), with respect to the free ion value by taking neutron diffraction measurements at 4.2 K [52].* *The covalency affects the orbital magnetic moment as well as the spin–orbit interaction [49] since it is the coupling between the spin and the orbital angular momenta of the atom [3].*

**Evidence 3.**  *The presence of covalency can cause a redistribution of the charge and spin densities over the d-transition metals and the ligands in the material. Evidence 3 is also a consequence of Evidence 1 and 2 of this section.*

**Explanation 3.**  *Covalency generally induces a redistribution of the charge and spin densities from the metal ion (cation) to the ligands (anions) [51].* *Equations (1) and (2) show these quantities mathematically.*

(1) $$charge\;density=n_{↑}+n_{↓}$$

(2) $$spin\;density=n_{↑}-n_{↓}$$

This effect, like the previous ones, can be experimentally investigated through neutron diffraction studies [48,53,54]. The spin density is never completely cancelled out in the ligands [48]. However, the spin density distribution may be affected by the presence of spin polarization [45,48,51]. The difference between these two quantities is that spin polarization does not possess a positive or a negative sign with respect to the spin density (see Equation (2)) as shown in Equation (3) [3].

(3) $$spin\;polarization=\left|\frac{n_{↑}-n_{↓}}{n_{↑}+n_{↓}}\right|$$

Spin polarization is associated with the presence of an excess of either spin orientation. Since it is known that the exchange interaction [55,56] is responsible for lowering the energy of orbitals with the same spins [57], the total spin polarization will create a difference between the spin up and spin down orbitals in terms of the spin distribution on metal ions and ligands. Since *d*-transition metal ions are responsible for carrying the unpaired electrons, the spin density will be higher on metals than on ligands [49,51]. Consequently, the spin polarization can also induce a redistribution of magnetic moments on metal ions and ligands [49,51]. An interesting example of this is the role played by the spin polarization in the reduction of Cr^3+^ (3d^3^) magnetic moment in G-type AFM lanthanum chromium perovskite (LaCrO_3_), experimentally investigated by neutron diffraction studies [51,52]. This effect should be considered while investigating the chemical bonding in collective and, more in general, high-spin magnetic materials [48,51]. Indeed, the influence of the spin polarization on the redistribution of spin density has been experimentally reported in the case of materials containing high-spin 3d^5^ (e.g., Mn^2+^) and 3d^3^ (e.g., Cr^3+^ and Mn^4+^) ions [45,48,51]. In general, experimental spin density measurements can help us estimate the amount of covalency in a material [45].

**Evidence 4.**  *Covalency can affect magnetic interactions in magnetic materials, particularly in 3d-metal-based oxides.*

**Figure 1:**
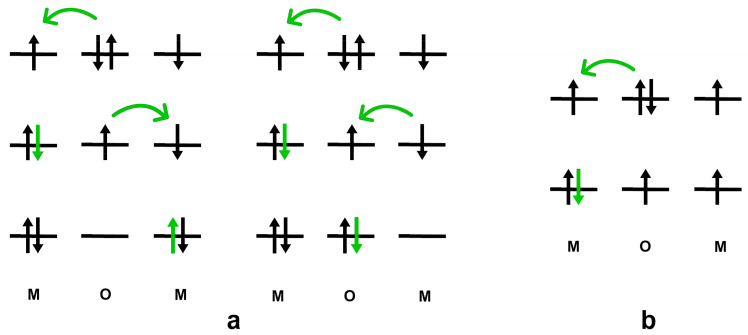
Simple sketch of the super-exchange mechanism in magnetic oxides. The hopping of electrons (green arrow) is described in the case of antiparallel (**a**) and parallel (**b**) spins on the *d*-transition metal orbitals.

**Explanation 4.**  *The most investigated materials for the interplay between covalency and magnetism are magnetic oxides [11,49,51,58].* *Among magnetic oxides, the 3d-metal ones are of particular interest since the participation of the d-electrons in both their chemical bonding and magnetic phenomena is more evident in these oxides [11].* *Some 3d-orbitals of the metals, together with the 2p-orbitals of the ligands, participate in chemical bonds by orbital overlapping (i.e., bonding orbitals), while the remaining ones (i.e., antibonding orbitals) host unpaired electrons that are responsible for their magnetic properties [11,50,51].* *Moreover, the 4s- and 4p-orbitals of d-transition metals can overlap with the ligand orbitals as well, thus participating in the bonding [51].* *Bonding orbitals are energetically more stable than antibonding ones. For example, J.B. Goodenough and A.L. Loeb suggested that the increment in the magnetic ordered–disordered transition temperature observed in rock-salt manganese monoxide (MnO), iron monoxide (FeO), cobalt monoxide (CoO), and nickel monoxide (NiO), with NiO (523 K) > CoO (291 K) > FeO (198 K) > MnO (122 K), is proof of the involvement of covalency in magnetic interactions, since covalency stabilizes the structure of such materials [58].* *Moreover, covalency can influence the energy difference between bonding and anti-bonding orbitals by forming an energy discrepancy that affects the conduction properties of the materials. This also helps to explain why covalent solids are poor conductors (see Table 1) [11,51].* *The unpaired electrons occupying the antibonding orbitals, instead, participate in magnetic phenomena through exchange mechanisms (including double-exchange and super-exchange mechanisms) [3,9].* *The most investigated exchange mechanism related to covalency is the super-exchange mechanism [11,49,50,51].* *In the super-exchange mechanism, the effective exchange interaction between two magnetic metals occurs through the mediation of non-magnetic atoms (e.g., oxygen in oxides) [59]*. *Figure 1* *shows a schematic picture of this mechanism, in which the d-orbitals of the d-transition metals possess only one unpaired electron each for simplicity. The super-exchange mechanism is a long-range interaction regulated by the occupation of d-transition metal orbitals (i.e., electron–electron Coulomb repulsion) and by the amplitude of the angle between the two neighbouring magnetic metals and the non-magnetic atom [59].* *Therefore, the very same p-orbitals of the ligand involved in the exchange mechanism also participate in the chemical bonding with the d-transition metal ions [59].* *Thus, the exchange interactions between neighbouring magnetic metals through non-magnetic atoms (e.g., oxygen atoms) are associated with covalency [49,58].* *Indeed, P.W. Anderson proposed that the long-range character of the super-exchange mechanism is due to the covalent binding effects with the core electrons* *[50].*

These four pieces of evidence show that both covalency effects and magnetic effects should be included to obtain a complete description of the nature of chemical bonding in materials. This is valid for all transition metals from 3*d*-elements (typically considered to be less covalent and more magnetic due to their less spatially extended orbitals [40,45]) to 4*d*- and 5*d*-elements, which are commonly reported to be more covalent and less magnetic due to their more spatially extended orbitals [40,45].

### 2.3. Experimental Evidence on Magnetism–Covalent Bonding Interplay during Chemisorption

So far, four pieces of experimental evidence on the relationship between magnetism and heterogenous catalysis have been provided to serve as a background for this next section. Here, several proofs on this interplay will be described for the chemisorption process. The chemisorption process has been chosen since, among all the steps of a catalytic transformation, it represents one of its first steps (or the first one) and so it can regulate its fulfilment. Moreover, the occurrence of a chemisorption process indicates that (at least) one or more chemical interactions happen on the catalyst surface. Thus, the information provided here may also be valuable in the understanding of adsorbate–catalyst surface interactions, a topic of great interest in heterogeneous catalysis.

**Evidence 1.**  *The magnetic susceptibility (* *χ* *) of a catalyst containing d-transition metal elements changes during chemisorption processes [12,60,61,62,63].*

**Figure 2:**
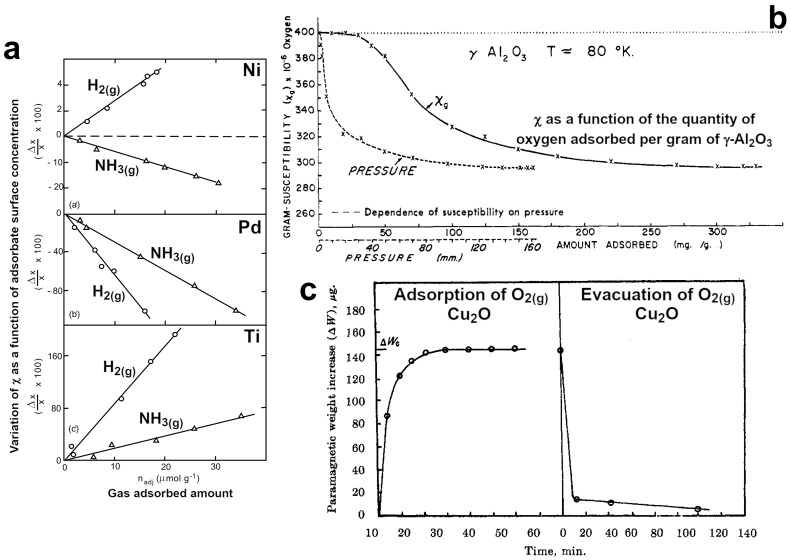
Examples of the variation in magnetic susceptibility during the chemisorption of various gasses. (**a**) Change in χ upon adsorption of H_2_ and NH_3_ gases on Ni (FM), Pd (paramagnetic), and Ti (paramagnetic) metal powders at 298 K (adsorbate surface concentration recorded between 50 Pa and 4 kPa equilibrium pressures). Reprinted and adapted with permission from Ref. [71]. Copyright 1995 Springer Nature. (**b**) Decrement in O_2_ magnetic susceptibility as a function of O adsorbed amount and, at the same time, of pressure at constant T (77 K) in a γ–alumina (γ–Al_2_O_3_) (diamagnetic) powdered sample obtained via a Faraday microbalance. Reprinted and adapted with permission from Ref. [65]. Copyright 1968 American Chemical Society. (**c**) Increment in χ during oxygen adsorption (T = 295 K) and desorption (T = 450 K) on powdered cuprous oxide (Cu_2_O) (diamagnetic) measured using the Gouy method. Reprinted and adapted with permission from Ref. [62]. Copyright 1964 American Chemical Society.

**Explanation 1.**  *Magnetic susceptibility (χ) measurements were the first experimental approach used to investigate the role of magnetism in catalytic reactions in the middle of the 20th century [1,12,63,64,65].* *Techniques based on χ* *measurements in solids, such as the Faraday and Gouy methods [12], proved to be useful in the characterization and investigation of paramagnetic and magnetic catalysts [1,12,13,65,66,67].* *For examples, they can be employed to distinguish between paramagnetism and collective behaviour [3,40], to identify magnetic transition temperatures [3,40], to measure changes in the oxidation state of the transition metal components of a catalyst [12,63,68,69], and* *to estimate the number of transition metal atoms involved in the chemisorption process [60,61].* *The use of these techniques is particularly interesting to investigate the adsorption of molecules over solid catalysts and to understand the nature of the interactions between the adsorbate and the solid catalyst. It has been experimentally reported that the adsorption of gas molecules, such as dihydrogen (H_2_), dioxygen (O_2_), carbon monoxide (CO), and organic substances (e.g., hydrocarbons), over a paramagnetic catalyst causes a reduction in the magnetic susceptibility of the transition metal atoms on the surface and in the total χ* *in the whole catalyst [60,61,70].* *This reduction was associated with the formation of bonds (i.e., the pairing of electrons) between the chemisorbed molecules and the surface atoms of the catalyst [61,70].* *This explanation is in agreement with Evidence 1 of the previous section and with the previously reported second rule of L. Pauling. Figure 2a shows the variation in magnetic susceptibility in the case of H_2(g)_ and ammonia (NH_3(g)_) adsorption over ferromagnetic (FM) Ni, paramagnetic palladium (Pd), and titanium (Ti) [71].* *The change in magnetic susceptibility during H_2(g)_ and NH_3(g)_ adsorption over palladium (Pd) is in agreement with the already reported trends for supported palladium (Pd) [60].* *However, the increment in χ in the case of H_2(g)_ over FM Ni and paramagnetic titanium (Ti) is unexpected and deserves a more in-depth investigation for a better understanding of the adsorbate–catalyst surface interactions. A possible explanation is provided by Evidence 2 of this section regarding the chemisorption of H_2_ on Ni catalysts. Conversely, an increment in the magnetic susceptibility has been observed in the case of the chemisorption of paramagnetic molecules (e.g., O_2(g)_) over diamagnetic catalysts. Figure 2c shows the variation in χ in diamagnetic cuprous oxide (Cu_2_O) during oxygen chemisorption [62].* *In this case, the increment is explained by the formation of paramagnetic species on the catalytic surface (e.g., Cu^2+^) [62].* *The magnetic susceptibility decreases instead when oxygen is almost or completely desorbed from the surface of the catalyst. Figure 2b describes the variation in O_2(g)_ magnetic susceptibility when the gas is adsorbed over diamagnetic gamma-alumina (γ-Al_2_O_3_) [65].* *The decrement in O_2(g)_ χ is in agreement with the formation of diamagnetic species (e.g., O^2-^ (ads)) over the diamagnetic catalyst [65].*

**Evidence 2.**  *The chemisorption of gas molecules (e.g., O_2_, H_2_, and CO) is associated with a variation in the magnetization at the surface with respect to its bulk value as well as the total magnetization of the solid catalyst [72,73].* *The change in the adsorbent magnetization can be experimentally seen only if substantial electronic interactions occur at the catalyst surface and if the number of metal atoms at the surface is high (i.e., it has a great “surface to volume” ratio). For this reason, modifications of the catalyst magnetization associated with adsorption processes were investigated in small metal nanoparticles with a diameter that was usually smaller than 10 nm [72]. A useful tool to study this piece of evidence from a theoretical point of view is the Newns–Anderson–Grimley chemisorption model coupled with the Stoner model of ferromagnetism [74].*

**Figure 3:**
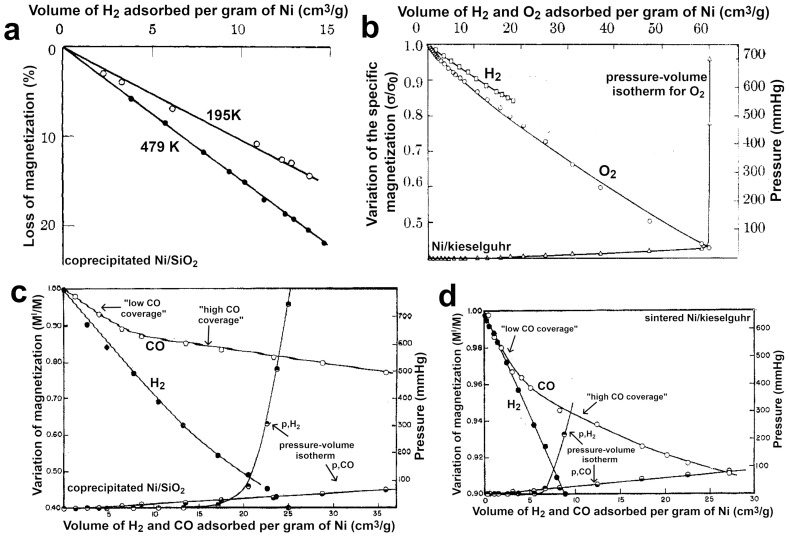
Examples of magnetization–volume isotherms for several gas molecules adsorbed at standard temperature and pressure (STP) conditions (T = 298 K and p = 1 atm) over FM Ni catalysts, obtained with a low-frequency AC permeameter and via volumetric gas adsorption techniques. (**a**) Magnetization–volume isotherm for H_2(g)_ on coprecipitated nickel/silica (Ni/SiO_2_) catalyst (37.5% of Ni) at 195 K and 479 K. The two isotherms were obtained under similar conditions (apart from the temperature). The change in magnetization is reported as percentage of loss of magnetization in the catalyst during the adsorption process Reprinted and adapted with permission from [75]. Copyright 1961 American Chemical Society. (**b**) Magnetization–volume isotherms for O_2(g)_ and H_2(g)_ on unsintered nickel/kieselguhr (52.8% Ni) at 298 K. The variation in the magnetization is measured using the specific magnetization or the mass magnetization (σ), the magnetization of the material per unit of mass. The pressure–volume isotherm for O_2(g)_ is also shown. Reprinted and adapted with permission from [76]. Copyright 1960 American Chemical Society. (**c**) Magnetization–volume isotherms for H_2(g)_ and CO_(g)_ on coprecipitated nickel/silica (Ni/SiO_2_) catalyst (~30% Ni) at 297 K under the same conditions. The diameter of the Ni particles is estimated at 2.5 nm. The corresponding pressure–volume isotherms are also shown. Reprinted and adapted with permission from [77]. Copyright 1962 American Chemical Society. (**d**) Magnetization–volume isotherms for H_2(g)_ and CO_(g)_ on sintered nickel/kieselguhr (~52% Ni) at 297 K under the same conditions. The catalyst has been sintered for 2 h at 873 K to increase the particle diameter, which is estimated to be in the range of 6.4 nm. This particle diameter is close to the upper limit of the low-frequency AC permeameter. The particle volume is around 15 times bigger than the one of the coprecipitated Ni/SiO_2_ displayed in (**c**). The corresponding pressure–volume isotherms are also shown. Reprinted and adapted with permission from [77]. Copyright 1962 American Chemical Society. In (**c**,**d**), the magnetization–volume isotherm for CO is divided into two sections, “low coverage” and the “high coverage”, according to the change in the isotherm slopes.

**Explanation 2.**  *This piece of evidence was experimentally observed in 3d-transition metals like FM Fe, Co, and Ni nanoparticles due to their spontaneous magnetization and high number of exchange interactions [72].* *Magnetic methods based on in situ measurements of the catalyst magnetization variation (i.e., the magnetic moment per volume of catalyst unit [3]) per adsorbed molecule of adsorbate were employed to investigate the adsorption of molecules and their binding on solid catalysts [72].* *The Weiss extraction method and low-frequency alternating current (AC) permeameters were employed for this purpose. In the former, uniform magnetic fields are used with a much higher strength than 1T (e.g., 100T), while in the latter low magnetic fields are applied (e.g., lower than 0.01–0.03 T). In both methods, the determination of the actual amounts of adsorbent and adsorbate is of primary importance to obtain reliable data. Methods based on magnetization measurements usually, yet not necessarily, require the sample to exhibit superparamagnetic behavior [3] under the experimental conditions [72]. Nevertheless, meaningful data are better extrapolated if the samples are actually superparamagnetic, especially when low-frequency AC permeameters are employed [72].* *A decrement in the magnetization is commonly observed when adsorbates like H_2_, O_2_, CO, and organic molecules (e.g., benzene and ethene) are chemisorbed on Fe, Co, and Ni catalysts. The results obtained from magnetization measurements can be expressed by using so-called magnetization–volume isotherms, in which the change in the catalyst magnetization is plotted against the volume of adsorbed molecule per gram of metal [72].* *Several studies on this topic can be found in the literature, for example, in the book titled “Chemisorption and Magnetization” by P.W. Selwood [72].* *Figure 3 shows four examples of magnetization–volume isotherms for the adsorption of various gasses on Ni catalysts obtained with a low-frequency AC permeameter. Figure 3a displays the isotherms for the hydrogen adsorbed at 298 K on a nickel/silica (Ni/SiO_2_) catalyst containing 37.5% Ni [75].* *The measurements are carried out at two different temperatures, 195 K and 479 K, in order to show the dependency of the hydrogen adsorption on the temperature when Ni is the adsorbent [72,75].* *Figure 3b displays the magnetization–volume isotherms of oxygen and hydrogen on a nickel/kieselguhr catalyst with 52.8% Ni at 298 K [76]. The pressure–volume isotherm for O_2(g)_ is also displayed since it is recorded simultaneously with the magnetization–volume one. The trends are similar for the two adsorbates up to 1 atm and at 298 K. However, in some cases, an increment in the magnetization is reported when hydrogen and oxygen are adsorbed on Ni catalysts (see also the increment in χ for the adsorption of hydrogen on Ni in Figure 2a as a further, related example). A possible explanation might be that the samples do not exhibit superparamagnetism under the experimental conditions due to a bigger particle diameter [72,76]. It is worth mentioning that the investigation of oxygen adsorption represents a challenge in this field due to the difficulty in distinguishing between real chemisorption and bulk oxidation [72,76]. Figure 3c,d display the magnetization–volume isotherms of hydrogen and CO on two different Ni catalysts, nickel/silica and nickel/kieselguhr, both at 297 K [77]. The most important difference between these two catalysts is the particle size, with the catalyst in Figure 3d being ~two and a half times larger than the one in Figure 3c. CO isotherms are particularly interesting since they show how the CO adsorption mode changes with the particle size of the catalyst and with its coverage [72,77]. Moreover, the change in the slope of the magnetization–volume isotherms for hydrogen agrees with the dependency of the hydrogen adsorption on the particle size of the Ni catalysts described in Figure 3b.*

**Evidence 3.**  *The chemisorption of gas molecules (e.g., O_2_, H_2_, and CO) is associated with a change in the magnetic moment(s) of the surface atoms of a catalyst [78,79].* *The effect has been experimentally observed in magnetic surfaces and films containing FM 3d-metals such as Fe, Co, and Ni. Several authors have also reported a change in the magnetic moment of the adsorbate due to the chemisorption process [78,80,81]. This is related to the previous one and to Evidence 2 of the previous section.*

**Explanation 3.**  *The change in the magnetic moments on the surface atoms of the catalyst and the adsorbate due to chemisorption can be investigated by several techniques based on spin-polarized electrons: for example, spin-polarized photoemission studies [82], spin-resolved inverse photoemission spectroscopy [78], spin-polarized inverse photoelectron spectroscopy (SPIPES) [83], spin-polarised Auger electron spectroscopy (SPAES) [80], and spin-polarized secondary electron microscopy (SP-SEM) [84].* *A reduction in the magnetic moment(s) of 3d-metal atoms on the surface is generally reported as the result of the adsorption of molecules over the ferromagnetic surface [78,79].* *Generally, this effect is associated with the formation of bonds (i.e., the pairing of electron spins) between the adsorbates and the catalyst surface atoms. The interaction usually involves the p- or s-electrons of the adsorbate and the d-electrons of the transition metals of the catalyst surface [85,86].* *In some cases, the bond can be so strong (i.e., possessing a high covalent character) that the magnetic moments on the surface atoms involved in the chemisorption are almost or completely cancelled out. In the latter case, it is said that an induced “magnetically dead layer” is formed since the atoms at the catalyst surface do not possess unpaired electrons and do not participate further in cooperative magnetic phenomena [87].* *For example, adsorbate-induced “dead layers” have been experimentally observed when O atoms are chemisorbed over Ni(110) via spin-resolved photoemission studies [79,88].* *Conversely, the magnetic moments of Ni atoms involved in the chemisorption process are not completely quenched when the adsorbate is CO, thus no “dead layers” are observed [79].* *Some authors have also proposed that these “dead layers” already exist on the catalyst surface without the presence of an adsorbate. It is claimed that their thickness depends on the preparation method and on the temperature [72].* *The possible presence of these induced “magnetically dead layers” due to the chemisorption of gas molecules is commonly referred as an “adsorbate-induced effect”. Conversely, the occurrence of magnetic moments in the adsorbate atoms due to magnetic coupling with FM 3d-metals on the surface is generally referred to as a “metal-induced effect”. Two connected phenomena should occur to observe changes in the adsorbate magnetic moment:*

(1) The orbital hybridization between the *sp*-electrons of the adsorbate and the *d*-electrons of the FM catalyst;
(2) The formation of additional bands (i.e., substantial exchange splitting) in the electronic structure of the adsorbate upon chemisorption over an FM surface [78,81,86,89].

Experimental proof of the “metal-induced effect” can be provided via spin-resolved photoemission spectroscopy or other techniques able to investigate the exchange splitting of the adsorbate bands upon chemisorption [78,81,90]. However, further work is still needed to completely comprehend the interactions between adsorbates and adsorbents.

**Evidence 4.**  *The formation of covalent bonds between the catalytic surface and the adsorbate during chemisorption can lead to catalyst poisoning [1].* *This piece of evidence is particularly related to the previous arguments of this section.*

**Explanation 4.**  *Resistance against poisoning is a relevant feature of a solid catalyst, since poisoning is one of the major origins of selectivity and/or activity losses [2].* *Catalyst poisoning can occur when an impurity present in the gaseous reactants strongly interacts with the active sites of the catalyst surface through covalent bonding [2,91].* *Poisons interact with the active sites of the catalyst by partially or completely deactivating them depending on their concentration in the reactor [91]. Table 4 reports notable poisons for few relevant industrial reactions.*

The poisoning of a solid catalyst can be experimentally detected and investigated through magnetic methods such as magnetic susceptibility, since it causes a change in the χ of the catalyst (see Evidence 1 above) [92]. These methods were employed to investigate the poisoning of palladium (Pd) by alkyl sulfides [70] and of nickel/silica (Ni/SiO_2_) by arsine (AsH_3_) [93] and other poisons, such as sulfuric acid (H_2_S), thiophenol (C_6_H_6_S), NH_3_, and pyridine (C_5_H_5_N) [92]. More recently, magnetic susceptibility measurements were taken to monitor the amount of Fe contamination in the fluid catalytic cracking process, showing several advantages, such as selectivity, fast results, robustness, and non-destructiveness [94].

**Evidence 5.**  *The spin polarization at the surface of an FM catalyst changes during the chemisorption of gas molecules such as O_2_ and H_2_ [79,95,96,97].* *Consequently, the adsorbate spin polarization can increase or decrease as well [79,95]. This piece of evidence is also a consequence of the previous ones and connected with Evidence 3 of the previous section.*

**Explanation 5.**  *This phenomenon was experimentally observed in FM catalysts such as Ni by using, for example, spin-resolved spectroscopic methods (e.g., spin-resolved photoemission and spin-polarized Auger electron and electron-capture spectroscopies) [80,95,97,98,99,100].* *In the case of Evidence 5, the experimentally accessible quantity is the spin polarization, instead of the spin density, as reported in Evidence 3 of the previous section. Generally, a reduction in the spin polarization of the catalytic surface and a concomitant increment in the adsorbate one is reported upon the chemisorption of molecules. For example, the effect was observed in the case of O-adsorption over Ni(110) [79,83], and H-adsorption over Ni(100) [96] and Ni(110) [100]. In contrast, the CO-adsorption over Ni(110) causes only a reduction in the spin polarization at the surface Ni atoms [79,101]. The same authors explain that the spin polarization of CO remains unaffected upon chemisorption since no experimentally appreciable exchange splitting is observed [79] (see Evidence 3 of this section). Evidence 5 is considered an “adsorbate-induced effect”.*

**Further evidence.**  It is also important to mention that other phenomena associated with the chemisorption of molecules over ferromagnetic surfaces have been experimentally reported besides those described above. For example, several authors have observed a change in magnetic anisotropy on the FM catalyst surface [76,98,102,103,104,105]. Techniques like electron paramagnetic resonance (EPR) [3,106,107], magnetic force microscopy [3,108], nuclear magnetic resonance (NMR) [3,109], the magneto-optical Kerr effect (MOKE) [104], X-ray magnetic circular dichroism (XMCD) [3,104], and spin-polarized low-energy electron microscopy (SPLEEM), coupled with an ultra-high vacuum (UHV) chamber [103], represent some useful tools to investigate this change. Other authors have reported a strong Dzyaloshinskii–Moriya interaction [3] induced by the chemisorption of oxygen on a FM Ni layer at room temperature via spin-polarized low-energy electron microscopy (SPLEEM) [110]. Others authors have observed a change in the electron correlation on the FM catalyst surface due to the adsorption of O atoms [111]. Another interesting example is the work of M. Kurahashi and Y. Yamauchi on O_2_ adsorption over an in-plane magnetized Ni(111) film carried out using spin- and alignment-resolved chemisorption techniques [112,113]. Their results show that the sticking probability of O_2_ over magnetized Ni(111) film is different with parallel and antiparallel spin orientations of the paramagnetic molecule, with respect to the majority of the spins’ direction on the catalyst surface. The preferred O_2_ spin orientation is the one antiparallel to the spin orientation of the FM catalyst. No such preference in the spin orientation is observed when the O_2_ is chemisorbed over the non-spontaneously magnetic (paramagnetic) W(110). It is worth mentioning that, besides the aforementioned magnetic and spectroscopic methods, neutron diffraction and scattering studies also represent useful tools to investigate the relationship between magnetism and heterogeneous catalysis, as shown in the previous section [114].

These pieces of evidence show that the inclusion of the magnetic changes (e.g., magnetic susceptibility, magnetization, and/or magnetic moments) of a catalyst upon chemisorption results in a proper, complete, and more exact description of this chemical event. The same statement was pointed out by D.A. Dowden [1]. This inclusion represents a step forward toward a better understanding of the relationship between magnetism and heterogenous catalysis.

## 3. Conclusions

The main requirements for a material to be employed as a solid catalyst in industrial catalytic processes are as follows: a high selectivity, a high stability under catalytic conditions, and a high activity. Identifying optimum catalytic compositions that possess all three desired requirements still represents a veritable challenge nowadays. Understanding the electronic, physical, chemical, and magnetic properties of solid catalysts themselves and during catalytic reactions is a starting point, but also a complex and multifaced end point in and of itself. Indeed, the relationship between magnetism and heterogeneous catalysis is still the object of a lot of debate. In this work, we focused on the interplay between magnetism and covalent bonding. Experimental proofs of this interplay have been provided throughout the years, both in solids and during the chemisorption process. These experimental evidences prove that this interplay is accountable for chemical, physical, magnetic, and catalytic modifications of solid catalysts, and thus, it should not be disregarded when looking for optimum catalytic compositions. Moreover, even if this interplay represents only one aspect of the relationship between magnetism and heterogenous catalysis, it reveals the active role of magnetism in catalytic processes. To this end, future developments within this field should include the design of specific experiments targeting the relationship between magnetism and heterogeneous catalysis in chemisorption processes and in catalytic activation steps. Such experiments, in synergy with computational investigations, may help provide an improved and more complete understanding of this topic.

## Figures and Tables

**Table 1:** Examples of materials with predominant ionic, covalent, metallic, or molecular binding and their characteristics.

Name	Chemical Bond (Strength)	Features	Examples
Ionic solids	Ionic (strong)	Close-packed structures Insulators Diamagnetic (if they do not contain transition metals)	NaCl, CsCl
Covalent solids	Covalent (strong)	Structures less efficiently packed Semiconductors (mainly) Diamagnetic	diamond, Si, Ge
Metals	Metallic (medium)	Close-packed structures High electrical conductivity Paramagnetic (exceptions: some late- and post-transition metals)	Na, Nb, Pt, Ir
Molecular solids	Hydrogen bonds, van der Waals interactions, hydrophobic interactions, London dispersion forces, dipole–dipole interactions (weak individually, but may be strong in numbers)	Close-packed structures (in general) Compositions consist of inert gasses (i.e., noble gasses) or molecular components Insulators with low melting points	crystalline Ar, solid HF, crystalline H_2_O

**Table 2:** Selected examples of solid catalysts used in industrially relevant catalytic processes and the corresponding operating conditions in which they are employed.

Catalytic Reaction	Catalyst	Operating Conditions	Ref.
Oxygen reduction reaction (ORR)	Platinum (Pt)	334–394 K (PEMFC) ^a^	[29]
	L1_0_ Platinum-Cobalt (PtCo)		[15,30]
Hydrogen evolution reaction (HER)	Platinum (Pt)	298–393 K, 1–200 bar	[31]
	Nickel-Molybdenum (Ni-Mo) alloys		
	Nickel (Ni), Nickel (Ni) foam		
Oxygen evolution reaction (OER)	Iridium dioxide (IrO_2_)	298–393 K, 1–200 bar	[31]
	Ruthenium dioxide (RuO_2_)		
	Cobalt nickel oxide (CoNi_2_O_4_)		
Ammonia (NH_3_) synthesis	Iron (Fe)-based catalysts	674–774 K, 100–450 bar	[32]
	Magnetite (Fe_3_O_4_), Potassium oxide (K_2_O), Alumina (Al_2_O_3_))	724–774 K, 250–450 bar	[2]
Hydrogenation of ketones/aldehydes to alcohols	Nickel (Ni)	374–424 K, 30 bar	[2]
	Platinum (Pt)		
	Copper (Cu)		
Methanol (CH_3_OH) synthesis	Zinc oxide-Chromic oxide (ZnO-Cr_2_O_3_)	524–674 K, 200–300 bar	[2]
Oxidation of ethene to epoxyethane (H_2_C=CH_2_ → C_2_H_4_O)	Silver (Ag)/support	474–524 K, 10–22 bar	[2]
Oxidation of methanol (CH_3_OH) to formaldehyde (CH_2_O)	Silver (Ag) crystalline	~874 K	[2]
Polymerization of ethene (H_2_C=CH_2_)	Chromic oxide/Silica (Cr_2_O_3_/SiO_2_)	324–424 K, 20–80 bar	[2]
	Chromic oxide/Molybdenum trioxide (Cr_2_O_3_/MoO_3_)		
Production of gasoline from cracking of kerosene	Alumina/Silica (Al_2_O_3_/SiO_2_) zeolites	774–824 K, 1–20 bar	[2]
Propene (H_2_C=CHCH_3_) epoxidation by dioxygen (O_2(g)_)	Copper (Cu)-based catalysts	474–673 K 0.01–0.5 bar	[33]
	Silver (Ag)-based catalysts		
Production of vinyl chloride from ethene (H_2_C=CH_2_) and hydrochloric acid/dioxygen (HCl/O_2_)	Cupric chloride/Alumina (CuCl_2_/Al_2_O_3_)	474–514 K, 2–5 bar	[2]

^a^ PEMFC= proton exchange membrane fuel cells.

**Table 3:** The determination of the electronic ground state of a material requires the solution of the Schrödinger equation ($\hat{H}\Psi =E\Psi$), in which the Hamiltonian operator ($\hat{H}$) encompasses various terms reported below together with energetic contributions acting on *d*-electrons. n is the principal quantum number.

Hamiltonian Operator					
$\hat{H}=T_{e^{-}}^{kinetic}+V_{N+e^{-}}^{Coul}+V_{e^{-}+e^{-}}^{Coul}+V_{L}+V_{s-o}$					
Energy contributions					
$T_{e^{-}}^{kinetic}$	kinetic energy of the electrons				
$V_{N+e^{-}}^{Coul}$	Coulomb attraction between nucleus and electrons				
$V_{e^{-}+e^{-}}^{Coul}$	electron–electron Coulomb repulsions				
$V_{L}$	crystal field potential				
$V_{s-o}$	energy contribution of the spin–orbit interaction				
	Strength of the Energy contributions (eV)			n	Order of Energy contributions
	$V_{e^{-}+e^{-}}^{Coul}$	$V_{L}$	$V_{s-o}$		
*nd*	3*d* > 4*d* > 5*d* ≈ 1.23 ($V_{e^{-}+e^{-}}^{Coul}+V_{N^{+}+e^{-}}^{Coul}$) = 1–10 (3*d*)	3*d* < 4*d* < 5*d* ≈ 2.48 ≈1.5 (3*d*)	3*d* < 4*d* < 5*d* ≈ 0.12 ≈0.1 for 3*d*	3*d* ^N^	$V_{e^{-}+e^{-}}^{Coul}\;$ ≈ $\;V_{L}\;$ > $V_{s-o}$ $V_{L}\;$ > $\;V_{e^{-}+e^{-}\;}^{Coul}$ > $V_{s-o}$ (for N = 6 (LS))
				4*d* ^N^	$V_{L}\;$ > $V_{e^{-}+e^{-}}^{Coul}\;$ > $V_{s-o}$
				5*d* ^N^	$V_{L}$ > $\;V_{e^{-}+e^{-}}^{Coul}\;\approx \;V_{s-o}$

**Table 4:** Examples of catalyst poisons for some industrial processes.

Catalytic Reaction	Catalyst Poisons	Refs.
Ammonia (NH_3_) synthesis	Strong: sulphur (S), carbon monoxide (CO), selenium (Se), tellurium (Te), phosphorus (P), halogens, arsenic (As)-containing compounds Weak: water (H_2_O), dioxygen (O_2_), nitric oxide (NO)	[2,91]
Hydrogenation of ketones/aldehydes to alcohols	Sulphur (S), selenium (Se), tellurium (Te), phosphorous (P), arsenic (As)-containing compounds, halogens, dioxygen (O_2_), carbon monoxide (CO)	[2]
Oxidation of ethene to epoxyethane (H_2_C=CH_2_ → C_2_H_4_O)	Acetylene (HC≡CH)	[91]
Oxidation of methanol (CH_3_OH) to formaldehyde (CH_2_O)	Iron (Fe), nickel (Ni), carbonyls	[91]
Production of gasoline from cracking of kerosene	Ammonia (NH_3_), sodium (Na), organic bases, heavy metals, coke	[2,91]

## References

[1] Dowden D.A. (1950). 56. Heterogeneous catalysis. Part I. Theoretical basis. J. Chem. Soc..

[2] Hagen J. (2005). Industrial Catalysis: A Practical Approach.

[3] Blundell S. (2001). Magnetism in Condensed Matter. Oxford Master Series in Condensed Matter Physics.

[4] Getzlaff M. (2008). Fundamentals of Magnetism.

[5] Wijn H.J. (1991). Magnetic Properties of Metals. d-Elements, Alloys and Compounds. Data in Science and Technology.

[6] Ryden W.D., Lawson A.W. (1970). Magnetic Susceptibility of IrO_2_ and RuO_2_. J. Chem. Phys..

[7] Mabbs F.E., Machin D.J. (2008). Magnetism and Transition Metal Complexes.

[8] Biz C., Fianchini M., Gracia J. (2021). Strongly Correlated Electrons in Catalysis: Focus on Quantum Exchange. ACS Catal..

[9] Biz C., Gracia J., Fianchini M. (2022). Review on Magnetism in Catalysis: From Theory to PEMFC Applications of 3d Metal Pt-Based Alloys. Int. J. Mol. Sci..

[10] Ishikawa Y.M.N. (1991). Physics and Engineering Applications of Magnetism. Springer Series in Solid-State Science 92.

[11] Goodenough J.B. (1963). Magnetism and the Chemical Bond.

[12] Selwood W. (1946). Magnetism and Catalysis. Chem. Rev..

[13] Voorhoeve R.J.H. (1974). Experimental Relationships between Catalysis and Magnetism. AIP Conf. Proc..

[14] Richardson J.T. (1978). Magnetism and catalysis. J. Appl. Phys..

[15] Cullen D.A., Neyerlin K.C., Ahluwalia R.K., Mukundan R., More K.L., Borup R.L., Weber A.Z., Myers D.J., Kusoglu A. (2021). New roads and challenges for fuel cells in heavy-duty transportation. Nat. Energy.

[16] Sapountzi F.M., Gracia J.M., Weststrate C.J., Fredriksson H.O.A., Niemantsverdriet J.W. (2017). Electrocatalysts for the generation of hydrogen, oxygen and synthesis gas. Prog. Energy Combust. Sci..

[17] Anantharaj S., Kundu S., Noda S. (2020). “The Fe Effect”: A review unveiling the critical roles of Fe in enhancing OER activity of Ni and Co based catalysts. Nano Energy.

[18] Luo S., Elouarzaki K., Xu Z. (2022). Electrochemistry in Magnetic Fields. Angew. Chem. Int. Ed..

[19] Ren X., Wu T., Gong Z., Pan L., Meng J., Yang H., Dagbjartsdottir F.B., Fisher A., Gao H.-J., Xu Z.J. (2023). The origin of magnetization-caused increment in water oxidation. Nat. Commun..

[20] Kittel C. (2005). Introduction to Solid State Physics.

[21] Rossler U. (2009). Solid State Theory: An Introduction.

[22] Miller G.J., Zhang Y., Wagner F. (2017). Chemical Bonding in Solids. Handbook of Solid State Chemistry.

[23] David R.L. (2005). The Madelung Constant and Crystal Lattice Energy. CRC Handbook of Chemistry and Physics, Internet Version.

[24] Bader R.F.W. (1990). Atoms in Molecules. A quantum theory. The International Series of Monographs on Chemistry.

[25] Darling A.S. (1963). Cobalt-Platinum Alloys. A Critical Review of their Constitution and Properties. Platin. Met. Rev..

[26] Shi Q., Zhu C., Du D., Lin Y. (2019). Robust noble metal-based electrocatalysts for oxygen evolution reaction. Chem. Soc. Rev..

[27] Berlijn T., Snijders P.C., Delaire O., Zhou H.D., Maier T.A., Cao H.B., Chi S.X., Matsuda M., Wang Y., Koehler M.R. (2017). Itinerant Antiferromagnetism in RuO_2_. Phys. Rev. Lett..

[28] Gracia J., Biz C., Fianchini M., Amthor S. (2023). How advances in theoretical chemistry meet industrial expectations in electrocatalysts for water splitting. Catal. Sci. Technol..

[29] Alaswad A., Omran A., Sodre J.R., Wilberforce T., Pignatelli G., Dassisti M., Baroutaji A., Olabi A.G. (2021). Technical and Commercial Challenges of Proton-Exchange Membrane (PEM) Fuel Cells. Energies.

[30] Wang X.X., Swihart M.T., Wu G. (2019). Achievements, challenges and perspectives on cathode catalysts in proton exchange membrane fuel cells for transportation. Nat. Catal..

[31] Grigoriev S.A., Fateev V.N., Bessarabov D.G., Millet P. (2020). Current status, research trends, and challenges in water electrolysis science and technology. Int. J. Hydrogen Energy.

[32] Ponikvar Ž., Likozar B., Gyergyek S. (2022). Electrification of Catalytic Ammonia Production and Decomposition Reactions: From Resistance, Induction, and Dielectric Reactor Heating to Electrolysis. ACS Appl. Energy Mater..

[33] Teržan J., Huš M., Likozar B., Djinović P. (2020). Propylene Epoxidation using Molecular Oxygen over Copper- and Silver-Based Catalysts: A Review. ACS Catal..

[34] David. R.L. (2005). Magnetic susceptibility of the elements and inorganic compounds. CRC Handbook of Chemistry and Physics.

[35] Coey J.M.D., Venkatesan M., Xu H., Ogale S., Venkatesan T., Blamire M. (2013). Introduction to Magnetic Oxides. Functional Metal Oxides.

[36] Liu G., Hagelin-Weaver H., Welt B. (2023). A Concise Review of Catalytic Synthesis of Methanol from Synthesis Gas. Waste.

[37] Twigg M.V., Spencer M.S. (2001). Deactivation of supported copper metal catalysts for hydrogenation reactions. Appl. Catal. A Gen..

[38] Millar G.J., Collins M. (2017). Industrial Production of Formaldehyde Using Polycrystalline Silver Catalyst. Ind. Eng. Chem. Res..

[39] Pu T., Tian H., Ford M.E., Rangarajan S., Wachs I.E. (2019). Overview of Selective Oxidation of Ethylene to Ethylene Oxide by Ag Catalysts. ACS Catal..

[40] Mugiraneza S., Hallas A.M. (2022). Tutorial: A beginner’s guide to interpreting magnetic susceptibility data with the Curie-Weiss law. Commun. Phys..

[41] Hatscher S., Schilder H., Lueken H., Urland W. (2005). Practical guide to measurement and interpretation of magnetic properties. IUPAC Tech. Rep..

[42] Gothe M.L., Silva K.L.C., Figueredo A.L., Fiorio J.L., Rozendo J., Manduca B., Simizu V., Freire R.S., Garcia M.A.S., Vidinha P. (2021). Rhenium—A Tuneable Player in Tailored Hydrogenation Catalysis. Eur. J. Inorg. Chem..

[43] Luo L., Li H., Peng Y., Feng C., Zeng J. (2018). Rh-Based Nanocatalysts for Heterogeneous Reactions. ChemNanoMat.

[44] Krajczewski J., Ambroziak R., Kudelski A. (2022). Formation and selected catalytic properties of ruthenium, rhodium, osmium and iridium nanoparticles. RSC Adv..

[45] Tofield B.C. (1976). Covalency effects in magnetic interactions. J. Phys. Colloq..

[46] Su Y., Wang Z., Gao R., Wu Q., Zhao J., Zhu G., Li Q., Xu H., Pan Y., Gu K. (2023). Ferromagnetic L12-Pt3Co Nanowires with Spin-Polarized Orbitals for Fast and Selective Oxygen Reduction Electrocatalysis. Adv. Funct. Mater..

[47] Pauling L. (1931). The nature of the chemical bond. Application of results obtained from the quantum mechanics and from a theory of paramagnetic susceptibility to the structure of molecules. J. Am. Chem. Soc..

[48] Hubbard J., Marshall W. (1965). Covalency effects in neutron diffraction from ferromagnetic and antiferromagnetic salts. Proc. Phys. Soc..

[49] Owen J., Thornley J.H.M. (1966). Covalent bonding and magnetic properties of transition metal ions. Rep. Prog. Phys..

[50] Anderson W., Seitz F., Turnbull D. (1963). Theory of Magnetic Exchange Interactions:Exchange in Insulators and Semiconductors. Solid State Physics.

[51] Tofield B.C. (1975). The study of covalency by magnetic neutron scattering. Recent Impact of Physics on Inorganic Chemistry.

[52] Tofield B.C., Fender B.E.F. (1970). Covalency parameters for Cr^3+^, Fe^3+^ and Mn^4+^ in an oxide environment. J. Phys. Chem. Solids.

[53] Plakhty V.P., Gukasov A.G., Papoular R.J., Smirnov O.P. (1999). Spin density on ligands O^2−^ and covalency of Fe^3+^ ions in octahedral sites of the Ca_3_Fe_2_Ge_3_O_12_ garnet: A polarised neutron diffraction study. Europhys. Lett..

[54] Sawatzky G.A., Van Der Woude F. (1974). Covalency effects in hyperfine interactions. J. Phys. Colloq..

[55] Dirac A.M., Fowler R. (1997). On the theory of quantum mechanics. Proc. R. Soc. London. Ser. A Contain. Pap. A Math. Phys. Character.

[56] Heisenberg W. (1926). Mehrkörperproblem und Resonanz in der Quantenmechanik. Z. Phys..

[57] Szabo A.O., Ostlund N.S. (1996). Modern Quantum Chemistry. Introduction to Advanced Electronic Structure Theory.

[58] Goodenough J.B., Loeb A.L. (1955). Theory of Ionic Ordering, Crystal Distortion, and Magnetic Exchange Due to Covalent Forces in Spinels. Phys. Rev. B.

[59] Bluhm H.B.M.M., von Plessen G., Stampfer C. ( 2019). Electrons in solids. Graduate Texts in Condensed Matter.

[60] Candy J. (1983). On the use of magnetic methods for studying the adsorption of H_2_, O_2_, CO, and hydrocarbons on supported palladium. J. Chem. Soc. Chem. Commun..

[61] Candy J., Perrichon V. (1984). Magnetic study of CO and C2 hydrocarbons adsorption on PdSiO_2_ catalyst. J. Catal..

[62] Cotton J.D., Fensham P.J. (1964). Magnetic Susceptibility Changes during the Adsorption of Oxygen and Carbon Monoxide on Cuprous Oxide. J. Phys. Chem..

[63] Selwood W., Frankenburg W.G., Komarewsky V.I., Rideal E.K., Emmett P.H., Taylor H.S. (1951). Magnetism and the Structure of Catalytically Active Solids. Advances in Catalysis.

[64] Oikawa M. (1955). On the Relation between the Hydrogen Overpotential and the Magnetic Susceptibility of Copper-Nickel Alloys. Bull. Chem. Soc. Jpn..

[65] Mulay L.N., Mulay I.L. (1966). Magnetic Susceptibility: Recent Aspects of Instrumentation and Applications. Anal. Chem..

[66] Chernavskii P.A., Dalmon J.A., Perov N.S., Khodakov A.Y. (2009). Magnetic Characterization of Fischer-Tropsch Catalysts. Oil Gas Sci. Technol.-Rev. IFP.

[67] Morris H., Selwood P.W. (1943). Magnetic Measurements on Some Catalytically Active Substances. J. Am. Chem. Soc..

[68] Kadyk T., Eikerling M. (2015). Magnetic susceptibility as a direct measure of oxidation state in LiFePO_4_ batteries and cyclic water gas shift reactors. Phys. Chem. Chem. Phys..

[69] Rodulfo-Baechler S.M., González-Cortés S.L., Orozco J., Sagredo V., Fontal B., Mora A.J., Delgado G. (2004). Characterization of modified iron catalysts by X-ray diffraction, infrared spectroscopy, magnetic susceptibility and thermogravimetric analysis. Mater. Lett..

[70] Dilke M.H., Eley D.D., Maxted E.B. (1948). Catalytic Poisons and Magnetic Susceptibility. Nature.

[71] Stradella L., Genova E. (1995). The influence of chemisorbed gases on the magnetization of transition metals. J. Mater. Sci. Lett..

[72] Selwood W. (1975). Chemisorption and Magnetization.

[73] Gradmann U. (1991). Surface magnetism. J. Magn. Magn. Mater..

[74] Bhattacharjee S., Lee S.-C. (2021). Cooperation and competition between magnetism and chemisorption. Phys. Chem. Chem. Phys..

[75] Silvent J.A., Selwood P.W. (1961). The Mechanism of Benzene Chemisorption on a Supported Nickel Catalyst. J. Am. Chem. Soc..

[76] Leak R.J., Selwood W. (1960). The Chemisorption of Oxygen on Nickel. J. Phys. Chem..

[77] Besten I.E.D., Fox P.G., Selwood P.W. (1962). The mechanism of chemisorption: Carbon Monoxide and Carbon Dioxide on Nickel. J. Phys. Chem..

[78] Schönhense G., Donath M., Kolac U., Dose V. (1988). Exchange splitting of an oxygen 2p-derived band at Ni(100). Surf. Sci..

[79] Schmitt W., Hopster H., Güntherodt G. (1985). Influence of adsorbates on surface magnetism studied by spin-resolved photoemission spectroscopy. Phys. Rev. B.

[80] Sinković B., Johnson P.D., Brookes N.B., Clarke A., Smith N.V. (1989). Study of local magnetic properties of an adsorbate by spin-polarized Auger-electron spectroscopy. Phys. Rev. Lett..

[81] Johnson P.D., Clarke A., Brookes N.B., Hulbert S.L., Sinkovic B., Smith N.V. (1988). Exchange-Split Adsorbate Bands: The Role of Substrate Hybridization. Phys. Rev. Lett..

[82] Peter D.J. (1997). Spin-polarized photoemission. Rep. Prog. Phys..

[83] Seiler A., Feigerle C.S., Pena J.L., Celotta R.J., Pierce D.T. (1985). Connection between surface magnetism and electronic structure of oxygen on Ni(110). J. Appl. Phys..

[84] Nishihara K., Nomitsu T., Nakagawa T., Mizuno S. (2019). Structural investigation and magnetic properties of oxygen adsorption on ultrathin Fe(110) film. Surf. Sci..

[85] Johnson D. (1990). Magnetism and Chemisorption. J. Electron Spectrosc. Relat. Phenom..

[86] Abeledo C.R., Selwood W. (1962). Chemisorption of Hydrogen on Cobalt. J. Chem. Phys..

[87] Göpel W. (1979). “Magnetic dead layers” on chemisorption at ferromagnetic surfaces. Surf. Sci..

[88] Feder R., Hopster H. (1985). Chemisorption and surface ferromagnetism: Theoretical analysis of spin-resolved photoemission data from Ni(1 1 0)(2×1)−O. Solid State Commun..

[89] Dietz R.E., Selwood P.W. (1961). Effect of Chemisorbed Hydrogen on the Magnetization of Nickel. J. Chem. Phys..

[90] Donath M. (1989). Spin-resolved inverse photoemission of ferromagnetic surfaces. Appl. Phys. A.

[91] Forzatti P., Lietti L. (1999). Catalyst deactivation. Catal. Today.

[92] Martin G.A., Bartholomew C.H., Butt J.B. (1991). How Far Can Magnetic Methods Help in the Understanding of Metal Catalyst Deactivation?. Studies in Surface Science and Catalysis.

[93] Ng C., Chang Y. (1991). Arsine poisoning of nickel/silica catalysts : Hydrogen chemisorption study by magnetic method. Appl. Catal..

[94] Brandt S., Knöll J., Jiang H., Saar J., Fougret C. (2019). The Role of Magnetic Susceptibility in Detecting Iron Poisoning in FCC Equilibrium Catalyst Samples and Its Combination with Other Macroscopic Bulk Analysis Techniques. Ind. Eng. Chem. Res..

[95] Schmitt W., Kämper K.-P., Güntherodt G. (1987). Effect of adsorbates on the spin-polarized photoemission of itinerant ferromagnets. Phys. Rev. B.

[96] Landolt M., Campagna M. (1977). Demagnetization of the Ni(100) Surface by Hydrogen Adsorption. Phys. Rev. Lett..

[97] Feigerle C.S., Seiler A., Peña J.L., Celotta R.J., Pierce D.T. (1985). Chemisorbed oxygen on Ni(110) studied by spin polarized inverse photoemission. J. Vac. Sci. Technol. A.

[98] Elmers H., Gradmann U. (1988). Magnetometric analysis of the interaction of Ni(111) with oxygen. Surf. Sci..

[99] Allenspach R., Taborelli M., Landolt M. (1985). Oxygen on Fe(100): An Initial-Oxidation Study by Spin-Polarized Auger Spectroscopy. Phys. Rev. Lett..

[100] Rau C. (1982). Electron spin polarization esp at surfaces of ferromagnetic metals. J. Magn. Magn. Mater..

[101] Feigerle C.S., Seiler A., Peña J.L., Celotta R.J., Pierce D.T. (1986). CO Chemisorption on Ni(110): Effect on Surface Magnetism. Phys. Rev. Lett..

[102] Elmers H.J., Gradmann U. (1988). Surface magnetism of oxygen and hydrogen adsorption on Ni(111). J. Appl. Phys..

[103] Quesada A., Chen G., N'Diaye A.T., Wang P., Wu Y.Z., Schmid A.K. (2021). Non-monotonic magnetic anisotropy behavior as a function of adsorbate coverage in Fe ultrathin films near the spin reorientation transition. J. Mater. Chem. C.

[104] Ma X.-D., Nakagawa T., Yokoyama T. (2006). Effect of surface chemisorption on the spin reorientation transition in magnetic ultrathin Fe film on Ag(001). Surf. Sci..

[105] Chen G., Ophus C., Quintana A., Kwon H., Won C., Ding H., Wu Y., Schmid A.K., Liu K. (2022). Reversible writing/deleting of magnetic skyrmions through hydrogen adsorption/desorption. Nat. Commun..

[106] Meron S., Shenberger Y., Ruthstein S. (2022). The Advantages of EPR Spectroscopy in Exploring Diamagnetic Metal Ion Binding and Transfer Mechanisms in Biological Systems. Magnetochemistry.

[107] Bonke S.A., Risse T., Schnegg A., Brückner A. (2021). In situ electron paramagnetic resonance spectroscopy for catalysis. Nat. Rev. Methods Prim..

[108] Winkler R., Ciria M., Ahmad M., Plank H., Marcuello C. (2023). A Review of the Current State of Magnetic Force Microscopy to Unravel the Magnetic Properties of Nanomaterials Applied in Biological Systems and Future Directions for Quantum Technologies. Nanomaterials.

[109] Min S., Baek J., Kim J., Jeong H.J., Chung J., Jeong K. (2023). Water-Compatible and Recyclable Heterogeneous SABRE Catalyst for NMR Signal Amplification. JACS Au.

[110] Chen G., Mascaraque A., Jia H., Zimmermann B., Robertson M., Conte R.L., Hoffmann M., González Barrio M.A., Ding H., Wiesendanger R. (2020). Large Dzyaloshinskii-Moriya interaction induced by chemisorbed oxygen on a ferromagnet surface. Sci. Adv..

[111] Janas D.M., Droghetti A., Ponzoni S., Cojocariu I., Jugovac M., Feyer V., Radonjić M.M., Rungger I., Chioncel L., Zamborlini G. (2023). Enhancing Electron Correlation at a 3d Ferromagnetic Surface. Adv. Mater..

[112] Kurahashi M., Yamauchi Y. (2015). Spin Correlation in O_2_ Chemisorption on Ni(111). Phys. Rev. Lett..

[113] Kurahashi M. (2016). Oxygen adsorption on surfaces studied by a spin- and alignment-controlled O_2_ beam. Prog. Surf. Sci..

[114] Yu X., Cheng Y., Li Y., Polo-Garzon F., Liu J., Mamontov E., Li M., Lennon D., Parker S.F., Ramirez-Cuesta A.J. (2023). Neutron Scattering Studies of Heterogeneous Catalysis. Chem. Rev..

